# Promoter Hypermethylation-mediated Inactivation of *LRRC4 *in Gliomas

**DOI:** 10.1186/1471-2199-9-99

**Published:** 2008-11-03

**Authors:** Zuping Zhang, Dan Li, Minghua Wu, Bo Xiang, Li Wang, Ming Zhou, Pan Chen, Xiaoling Li, Shourong Shen, Guiyuan Li

**Affiliations:** 1 Cancer Research Institute, Central South University, Changsha 410078, Hunan, PR China; 2 Department of Parasitology, Central South University, Changsha 410078, Hunan, PR China; 3 The Third Affiliated Hospital of Xiang Ya School of Medicine, Central South University, Changsha 410078, Hunan, PR China

## Abstract

**Background:**

Leucine-rich repeat C4 protein (*LRRC4*) is a new member of the leucine-rich repeat (LRR) superfamily. It is not only a brain-specific gene but also a novel candidate for tumor suppression. *LRRC4 *inactivation is commonly found in glioma cell lines and primary glioma biopsies. However, little is known about the mechanism controlling *LRRC4 *expression. In a previous study, we did not find any genetic alteration in *LRRC4 *in primary glioma, which led us to explore an alternative mechanism underlying this phenomenon.

**Methods:**

In the present paper, we cloned the *LRRC4 *promoter with characteristics of a CpG island by luciferase reporter assay. Then, the CpG methylation status around the *LRRC4 *promoter region in glioma cell lines and primary gliomas was examined by methylation-specific PCR and bisulfite DNA sequencing. In order to demonstrate a functional association between *LRRC4 *promoter methylation and its gene inactivation, we performed DNA demethylation analysis with two human glioma cell lines using methylation-specific PCR and RT-PCR.

**Results:**

The sequence spanning positions -835 to -293 relative to the translation start site was identified as the LRRC4 promoter; this sequence is a TATA- and CAAT- less, high GC content region. It was found that *LRRC4 *promoter activity is strongly suppressed after treatment with SssI methylase in vitro. Furthermore, LRRC4 promoter methylation was observed by methylation-specific PCR in two glioma cell lines and all 30 primary glioma specimens, but not in normal brain tissue. Bisulfite DNA sequencing showed that most of the CpG sites were located around the *LRRC4 *promoter methylated in glioma cells and tissues, but not in normal brain tissue. In addition, the methylase inhibitor 5-Aza-2'-deoxycytidine could induce *LRRC4 *mRNA expression and *LRRC4 *promoter partial demethylation in SF126 and SF767 glioma cells.

**Conclusion:**

Methylation-mediated inactivation of *LRRC4 *is a frequent and glioma-specific event, and it may be a potential biomarker for diagnosis or prognosis, or serve as a therapeutic target.

## Background

Gliomas are the most common malignant tumors in the adult central nervous system and account for 50 to 60% of primary brain tumors. These cancers exhibit a relentless malignant progression characterized by widespread invasion throughout the brain, and thus usually result in a poor prognosis [1]. Although multiple genetic alterations are involved in the development and progression of malignant gliomas [2,3], epigenetic silencing of wild-type tumor suppressor genes via aberrant promoter hypermethylation has also been shown to occur [4-6]. Aberrant promoter methylation of CpG island-associated genes is a common epigenetic alteration associated with the inactivation of tumor suppressor and other genes in human cancers [7-9]. Unmethylated in normal tissues, promoters of these genes can become methylated *de novo *in cancer cells. This change is accompanied by alterations in histone modification and chromatin conformation, rendering the promoter transcriptionally inert [10].

Such epigenetic mechanisms have been implicated in the inactivation of several key regulators of the cell cycle (*RB, p16INK4A, p73*), DNA repair (*O*^6^*MGMT*), apoptosis (*DAP kinase*), angiogenesis (*THBS1*), and invasion (*TIMP3*) in glioma [11]. Recently, novel hypermethylated genes in glioma have been identified using a candidate gene approach or by a genome-wide screening method. The former revealed that genes such as EMP3, TMS1/ASC, SLC5A8, hMLH and PTEN are frequently targeted for DNA methylation-mediated silencing in glioma [4,5,12,13]. A genome-wide screen using a combined approach of pharmacologic inhibition of epigenetic modifications and gene expression microarrays also revealed that several novel genes are subject to aberrant hypermethylation in glioma [14,15]. Thus, aberrant methylation events have become critical to our understanding of the initiation and progression of human brain malignancies and may serve as a biomarker for diagnosis, prognosis and susceptibility to treatment.

Leucine-rich repeat C4 protein (*LRRC4*) is a new member of the leucine-rich repeat (LRR) superfamily located at 7q31-32 [16]. It was found to be predominantly expressed in normal brain tissue and involved in early nervous system development and differentiation [17], but the expression of *LRRC4 *was absent in several malignant glioma cell lines [18]. Similarly, it was absent or significantly down-regulated in 87.5% of primary glioma biopsies [16]. More importantly, *LRRC4 *had the potential to suppress tumorigenesis of U251 malignant glioma cells in vivo and cell proliferation in vitro [19]. Recent studies show that *LRRC4 *can block U251 cells in G_0_/G_1 _and induce U251 cell-growth arrest and differentiation by down-regulating the ERK/Akt/NF-κB, STAT3 and JNK2/p-c-Jun/p53 signaling pathways [18]. Therefore, the loss of *LRRC4 *function may be an important event in the progression of gliomas and may act as a novel candidate for tumor suppression. However, little is known about the mechanism of *LRRC4 *expression loss or down- regulation in glioma cell lines and biopsies. No known studies have found genetic alterations in the *LRRC4 *coding sequence in glioma biopsies or cell lines [18]. This lack of findings led us to explore an alternative mechanism underlying inactivation of *LRRC4 *in glioma.

In the present study, we cloned the *LRRC4 *promoter with characteristics of a CpG island. Then, CpG methylation status around the *LRRC4 *promoter region in glioma cell lines and primary gliomas was examined by methylation-specific PCR and bisulfite DNA sequencing. In order to demonstrate a functional association between *LRRC4 *promoter methylation and its gene inactivation, we performed DNA demethylation analysis with two human glioma cell lines using methylation-specific PCR and RT-PCR.

## Methods

### Cell lines and tumor samples

Thirty fresh tumor samples were collected after informed consent was obtained from patients who underwent brain operations for glioma at Xiangya Hospital (Hunan, People's Republic of China). The samples were snap-frozen immediately following resection and stored in liquid nitrogen until processing. The 17 male and 13 female patients were aged from 17 to 68 years (mean age at registration = 42 years). Tumors were graded and classified according to the World Health Organization (2007), including astrocytoma (grade I (1), grade II-III (17)), oligodendroglioma (grade II (5)), oligoastrocytoma (grade II (3)), and glioblastoma (grade IV (4)) [33]. For comparison, normal human tissues from patients without cancer were obtained at the time of autopsy.

Human glioblastoma-derived cell lines SF126 and SF767 were obtained from the Cell Research Institute of Peking Union Medical College (Peking, China) and cultured in minimal essential medium (MEM). Cos7 and Hela were obtained from American Type Culture Collection and maintained in Dulbecco's modified Eagle's medium (DMEM). All cells were supplemented with 10% heat-inactivated fetal bovine serum (FBS), 100 U/mL penicillin and 100 ug/mL streptomycin and cultured at 37C with 5% CO_2_.

### Cloning and analysis of the *LRRC4 *5' upstream regulatory region

The *LRRC4 *promoter region in the 5' end of humans was predicted using the PromoterInspector and PromoterScan programs. The CpG island was found using CpGplot from the European Molecular Biology Open Software Suite. To obtain the 5' flanking region of the *LRRC4 *gene, PCR amplification was performed on human genomic DNA (forward primer 5'-ATTGGTACCGGCGAGCTCACAGGGCAGGG-3', reverse primer 5'-CTTAGATCTCTGGAGAAGGAGGTGGGGAG-3'). After an initial denaturation step (94°C for 5 min), the PCR reactions were carried out for 32 cycles at 94°C for 30 sec, 62°C for 30 sec, and 72°C for 2 min, with a final extension of 10 min at 72°C. The PCR product was purified using a gel Purification Kit and cloned into the T/A cloning vector pGEMT-Easy (Promega). Positive clones of pT/A-2475/-101 were isolated and sequenced.

### Luciferase-reporter plasmid constructs and assay

The 5' upsteam regulatory region of the *LRRC4 *gene was subcloned into the KpnI and Bgl II restriction sites of the pGL3-enhancer vector (Promega). Construct naming is based on the position of the promoter fragments (pGL3-2475/-101). Four deletion constructs of the *LRRC4 *promoter region were created (pGL3-1483/-101, pGL3-835/-101, pGL3-293/-101 and pGL3-835/-293), originating from the construct pGL3 -2475/-101 by PCR amplification using the primers listed in Table 1. Sense primers for generating the reporter constructs described above contained an adaptor with a KpnI restriction site (GGTACC) at the 5' end. Anti-sense primers contained an adaptor with a Bgl II restriction site (AGATCT). The promoter fragments were then subcloned into the KpnI/Bgl II sites of the pGL3 enhancer vector and sequenced.

**Table 1:** Primer Pairs Used for Generating *LRRC4 *Promoter Constructs pGL3-2475/-101, pGL3-1483/-101, pGL3-835/-101, pGL3-293/-101 and pGL3-835/-293

pGL3-2475/-101	Forward: 5'-ATTGGTACCGGCGAGCTCACAGGGCAGGG-3'
	Reverse: 5'-CTTAGATCTCTGGAGAAGGAGGTGGGGAG-3'
pGL3-1483/-101	Forward: 5'-ATCGGTACCAGTATGCGTCAGCAGTACATTCACG-3'
	Reverse: 5'-CTTAGATCTCTGGAGAAGGAGGTGGGGAG-3'
pGL3-835/-101	Forward:5'-ATCGGTACCAACACCTCCTCTTAACTCTCGCC-3'
	Reverse: 5'-CTTAGATCTCTGGAGAAGGAGGTGGGGAG-3'
pGL3-293/-101	Forward:5'-ATTGGTACCTGCTTTCCTGCCTTCCTTCC-3'
	Reverse: 5'-CTTAGATCTCTGGAGAAGGAGGTGGGGAG-3'
pGL3-835/-293	Forward:5'-ATCGGTACCAACACCTCCTCTTAACTCTCGCC-3'
	Reverse: 5'-ATTAGATCTGCACTGGCGTGGTGTCCTTA-3'

Transfection was performed with Lipofectamine™ Reagent (Invitrogen, Carlsbad, CA). 5×10^5 ^cells were seeded in each well of 24-well tissue plates. When cells reached 50–80% confluence, they were cotransfected using 1 μg of each DNA construct in pGL3-enhancer and 0.25 μg SV40β-galactosidase vector for normalizing transfection efficiency per well according to manufacturer's instructions. Firefly luciferase activity was measured in cell lysates 48 h after transfection by using the Luciferase Assay System (Promega) and a luminometer. β-galactosidase activity was measured in cell lysates by the β-galactosidase Enzyme Assay System (Promega). Experiments were repeated at least three times with three replicates per sample for each experiment. Results are normalized against β-galactosidase activity.

### Methylation of report plasmid constructs in vitro

pGL3-835/-293 report plasmid constructs were methylated in vitro by SssI methylase (New England Biolabs) treatment following the manufacturer's instructions. Cells were transfected by methylated or mock-methylated constructs as described above.

### DNA extraction and Bisulfite modification

Genomic DNA from cells and tissues was prepared using a DNA Extraction Kit (TaKaRa) according to manufacturer's instructions. Five hundred nanograms of genomic DNA was modified and purified using an EZ DNA Methylation-Gold Kit (ZYMO RESEARCH), following the manufacturer's protocol. Modified DNA was used immediately or stored at -80°C for up to six months.

### Methylation-Specific PCR

The methylation-specific PCR primers were designed according to the promoter-active DNA sequence using Methyl Primer Express v1.0. Modified DNA was amplified by two different primer pairs specific to the unmethylated (U) and methylated (M) *LRRC4 *promoter sequences, respectively. For the methylated (M) sequence, the forward and backward primers were 5'-AGCGTAGTATTTAGCGAGTGC-3' and 5'-TAAACCCTAACACCGACTCG-3'.

For the unmethylated (U) sequence, the primers were forward 5'-GGGAGTGTAGTATTTAGTGAGTGT-3' and backward 5'-TAAACCCTAACACCAACTCACTC-3'. PCR amplification was performed for a total of 35 cycles with an annealing temperature of 58°C. Methylation specific PCR products were analyzed by a 2% agarose gel and stained with ethidium bromide.

### Bisulfite Sequencing

In order to cover the whole promoter region of *LRRC4*, two PCR regions were amplified using primers that avoided CpG sites. For Region 1, primers were 5'-GYGGATTGGAGAATTGATTT TT-3' and 5'-AACTATACAAATACATACCCCCCCC-3'. For Region 2, primers were 5'-GAGGGGGGGGTATGTATTTGTATAGT-3' and 5'-CCCACCCTCAAAACAAACCC TC-3'. The PCR amplification was performed for a total of 38 cycles with annealing temperature of 56°C. PCR products were gel-purified and cloned into the T/A cloning vector pGEMT-Easy (Promega). Ten subclones were isolated and identified by double digestion and sequencing.

### 5'-Aza-dC treatment of cell lines

The human glioblastoma-derived cell lines SF126 and SF767 were grown for 4 days in the presence of various concentrations of 5-Aza-dC (2.5, 5, 10 and 15 μM). Fresh drug was added every 24 h. RNA and DNA were separately isolated.

### RT-PCR

RNA was isolated from harvested cells with Trizol (Invitrogen) reagent and then treated with DNase (Roche) to eliminate contaminated DNA. Reverse transcription of the RNA was performed according to the instructions of Promega. To amplify *LRRC4*, 2 μl of cDNA was used for each PCR using the primers, 5'-CAACTTGGCCCACAAT AACC-3' (forward) and 5'-CATCCGACCCTCAGAAATGT-3' (reverse). The primers for GAPDH were 5'-GTCAGTGGTGGACCTGACC T-3' (forward) and 5'-AGGGGAGATTCAGTGTGGTG-3'(reverse). The GAPDH primers were added to the PCR at the end of the tenth cycle as control experiments. Ten microliters of each reaction was then run on 2% agarose gel and stained with ethidium bromide.

## Results

### Cloning and analysis of the 5' upstream regulatory region

**Figure 1:**
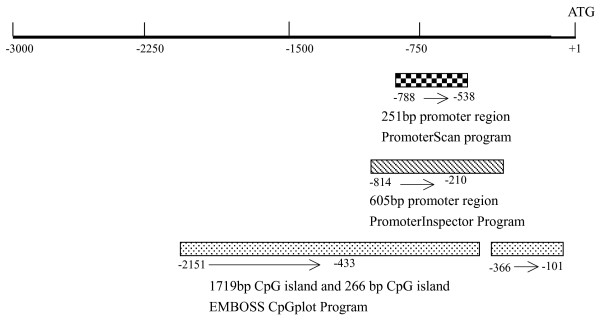
**Analysis of the *LRRC4 *5' upstream regulatory region by bioinformatics**. The regions showing the putative promoter activity and CpG islands are shown as a square box, a hatched box and dotted boxes, respectively. The translation start site is position +1 and the rest of the sequence is numbered relative to it.

To clone the *LRRC4 *promoter region, a database was searched against the human genomic DNA using *LRRC4 *(Genbank accession No. AF196976) as the query  to reveal the 5'upstream sequence of the *LRRC4 *gene. Several bioinformatics tools were used to identify the potential promoter region of the *LRRC4 *gene. A 605-bp region spanning positions -814 to -210 was identified as the potential promoter region of the *LRRC4 *gene by using a PromoterInspector [20], whereas a 251-bp region located from -788 to -538 was identified as the *LRRC4 *promoter by using a PromoterScan program [21]. We detected two CpG islands that spanned positions -2151 to -433 and -366 to -101 using the EMBOSS CpGplot program [22] (Figure 1). Finally, a genomic DNA fragment that spanned positions -2475 to -101 relative to the initiation codon ATG of the *LRRC4 *gene was amplified by PCR. The PCR product was cloned into the T/A cloning vector pGEMT-Easy (Promega) (Data not shown).

### Functional analysis of the *LRRC4 *promoter by 5' upstream deletion

To identify the promoter region of the *LRRC4 *gene, five constructs of progressive deletion spanning positions -2475 to -101 were generated (pGL3-2475/-101, pGL3 -1483/-101, pGL3-835/-101 and pGL3-293/-101) and cloned to upstream of the luciferase reporter gene in the pGL3-enhancer vector (Promega). Transient transfection experiments were carried out using Cos7 and Hela cells, respectively. The luciferase activity driven by *LRRC4 *promoter constructs was measured 48 h after transfection. Expression levels were corrected for variable transfection efficiencies by cotransfection with a plasmid directing the β-galactosidase expression.

**Figure 2:**
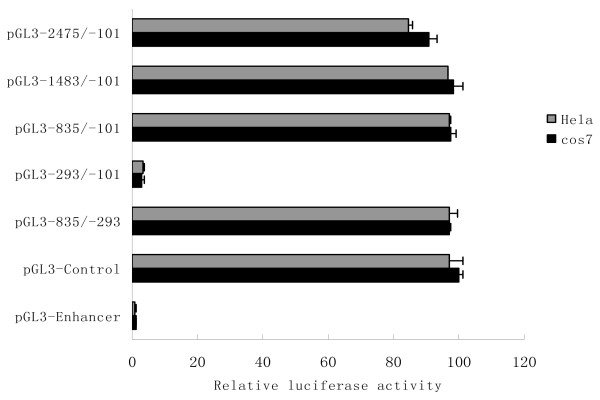
**Deletion analysis of the *LRRC4 *promoter**. Luciferase activity of the deleted constructs in Cos7 and Hela cells. Luciferase activity in Cos7 and Hela cells is represented by the black histogram and gray histogram, respectively. All transfection experiments were repeated at least three times, and luciferase activity was normalized by using β-galactosidase as an internal standard. Mean data ± standard errors are shown. In both cell lines, the construct from -835 to -293 was sufficient to mediate maximal promoter activity.

As shown in Figure 2, in Cos7 and Hela cells, luciferase expression driven by the constructs pGL3-2475/-101, pGL3-1483/-101, pGL3-835/-101 and pGL3-835/-293 exhibited similarly high levels, but the reporter driven by construct pGL3-293/-101 showed little luciferase activity. Thus, the sequence spanning position -835 to -293 relative to the translation start site of *LRRC4 *functions as a promoter. MethPrimer program analysis shows that the *LRRC4 *promoter region has high G/C content (approximately 70%) and characteristics of a CpG island [23]. In addition, this promoter region has no TATA box or CAAT box.

### DNA methylation suppresses *LRRC4 *promoter activity

**Figure 3:**
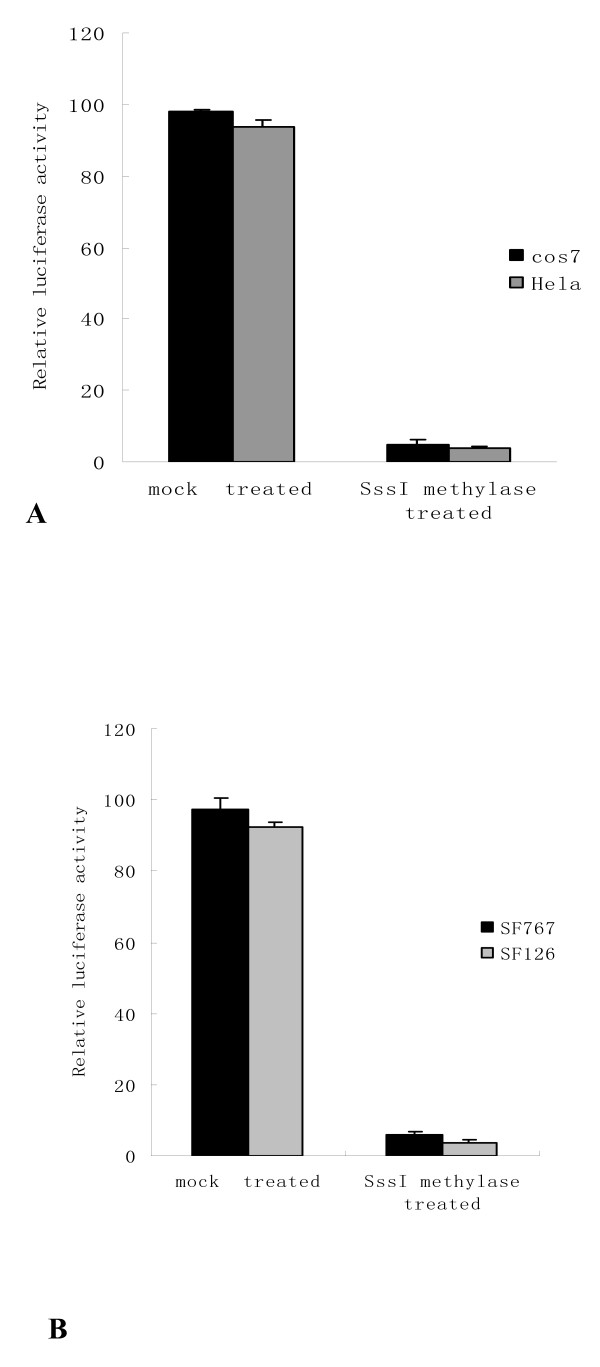
***LRRC4 *promoter activity is suppressed by methylation in vitro**. A. Luciferase activity assays showing *LRRC4 *promoter repression in response to methylation. Methylated (SssI) or mock-methylated *LRRC4 *promoter luciferase reporter constructs were transfected into Cos7 and Hela cells. Luciferase activity was measured in cell extracts 48 h after transfection. Luciferase activity in Cos7 and Hela cells is represented by the black and gray histograms, respectively. Luciferase activity was normalized using β-galactosidase as an internal standard. The results are shown as the mean ± SD of 3 independent experiments. B.*LRRC4 *promoter activity suppressed by methylation was verified in glioma cell lines SF126 and SF767.

The presence of a CpG island suggested the possibility that the gene might be regulated through changes in the methylation status, which have been shown to cause gene silencing [24,25]. To analyze the effects of DNA methylation on promoter activity, *LRRC4 *promoter reporter construct pGL3-835/-293 was treated with or without SssI methylase to methylate the promoter in vitro. In vitro-methylated or mock-methylated *LRRC4 *promoter constructs were transfected into Cos7 and Hela cells along with SV40β-galactosidase vector. Firefly luciferase activity was measured in cell lysates as shown in Figure 3A. It can be seen that *LRRC4 *promoter activity was strongly suppressed by methylation in both cell lines. The same result was verified in SF126 and SF767 cell lines (Figure 3B).

### Methylation of the *LRRC4 *promoter in glioma cell lines and biopsies

**Figure 4:**
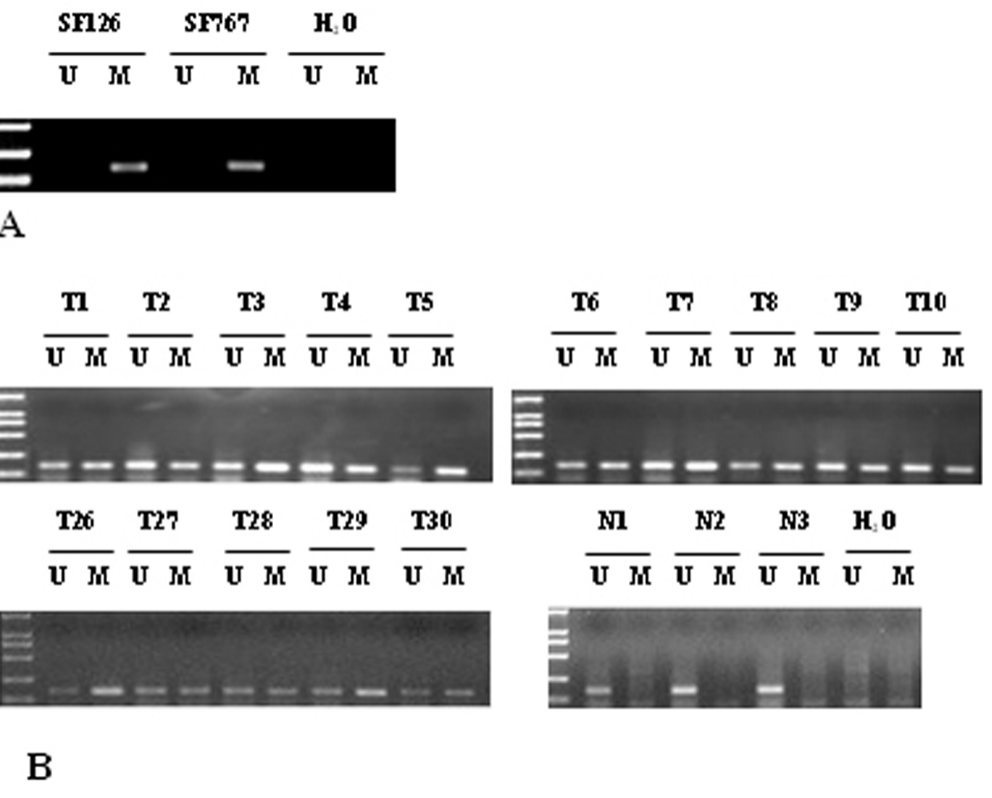
**Methylation of the *LRRC4 *promoter in glioma**. A. Methylation of the *LRRC4 *promoter in glioma cell lines SF126 and SF767. DNA from the indicated glioma cell lines was bisulfite-modified and analyzed for methylation of the *LRRC4 *promoter by methylation-specific PCR analysis. The presence of a PCR product in lane U indicates unmethylated *LRRC4*. The presence of a PCR product in lane M indicates the methylated *LRRC4*. B. Schematic presentation of methylation analysis of the *LRRC4 *gene promoter using MSP in tumor samples (T) taken from patients with glioma and three normal brain tissue samples (N).

Our previous study showed that the expression of *LRRC4 *was absent in glioma cell lines SF126 and SF767 [18]. Moreover, DNA sequence analysis failed to identify any mutation in the *LRRC4 *coding region, except for one Single Nucleotide Polymorphisms site hat does not affect amino acid sequences [18]. To clarify the mechanism of *LRRC4 *inactivation in these two cell lines, methylation-specific PCR was used to examine the methylation status of the *LRRC4 *promoter. Both SF767 and SF126 cell lines showed methylation of the *LRRC4 *promoter. The methylated sequence was obtained and is shown in Figure 4A.

To investigate whether aberrant methylation of the *LRRC4 *in glioma cell lines reflects an epigenetic event occurring in primary glioma, we next examined 30 primary glioma biopsies and three tissue specimens of normal brain for *LRRC4 *methylation. Some methylation analyses are shown in Figure 4B. It was demonstrated that the *LRRC4 *promoter was free from methylation in the three normal brain tissue samples, but was methylated to different extents in the 30 primary gliomas.

### Bisulfite sequence analysis of promoter region of *LRRC4*

**Figure 5:**
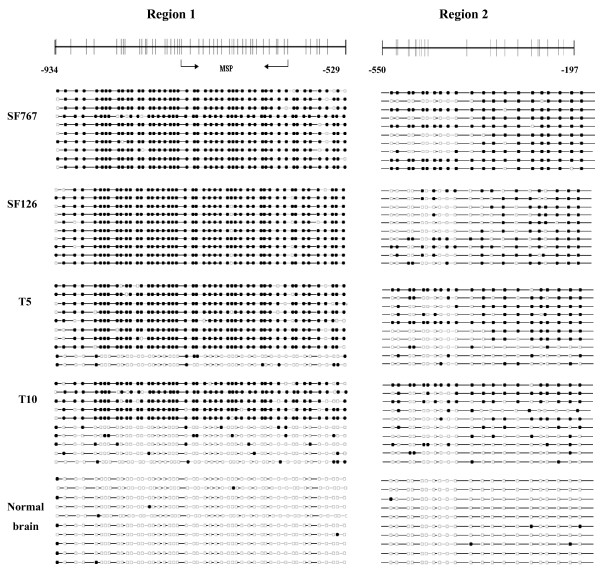
**Bisulfite sequence analysis of *LRRC4 *promoter**. Methylation status of CpG sites around the *LRRC4 *promoter region were analyzed in glioma cell lines (SF767 and SF126), primary glioma (T5 and T10) and normal brain tissue. Region 1 spans -934 to -529 relative to the *LRRC4 *translation start site, including 48 CpG sites. Region 2 spans -550 to -197 relative to the *LRRC4 *translation start site, including 21 CpG sites. The arrow represents the region amplified by methylation-specific PCR. Each row represents an individual subclone. Open circles represent unmethylated CpGs. Filled circles represent methylated CpGs.

To determine a more detailed map of the methylation in the *LRRC4 *promoter, we performed bisulfite sequencing around the promoter region of the *LRRC4 *gene in some of the glioma biopsies and cell lines studied above. In order to cover the whole promoter region of *LRRC4*, two PCR regions were amplified using primers that avoid CpG sites. Region 1 spans -934 to -529 relative to the *LRRC4 *translation start site, including 48 CpG sites. Region 2 covers -550 to -197 relative to the *LRRC4 *translation start site, including 21 CpG sites. Bisulfite sequencing of 10 individual clones of PCR products of both Region 1 and Region 2 from primary glioma biopsies (T5 and T10) and cell lines (SF126 and SF767) revealed densely methylated CpGs within the promoter regions compared to normal brain tissue (Figure 5). Whereas not all CpGs around the *LRRC4 *promoter are methylated, most of them are in SF126 and SF767 glioma cell lines. However, in the primary glioma tissue samples (T5 and T10), the methylation pattern appeared much more heterogeneous and varied in density in different clones of the same sample. This may be due to mixed cellularity in tissue samples.

### 5-Aza-dC induced the expression of *LRRC4 *in glioma cell lines

**Figure 6:**
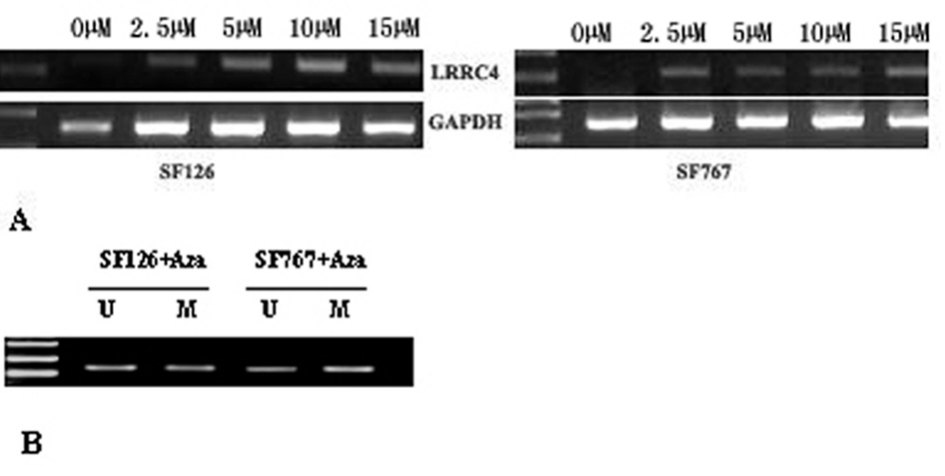
**5-Aza-dC induced the expression of *LRRC4 *in glioma cell lines**. A. Glioma cell lines SF767 and SF126 were treated with five different doses of 5-Aza-Dc (0, 2.5, 5, 10 and 15 μM), as indicated above each lane, for four days. Total RNA was isolated from control, and treated cells were then analyzed by RT-PCR using the *LRRC4 *primer set (top panel) and GAPDH primer set (bottom panel). B. Glioma cell lines SF767 and SF126 were treated with 5 μM 5-Aza-dC for four days. DNA was isolated and bisulfite-modified, and then analyzed for methylation of the *LRRC4 *promoter by methylation-specific PCR. The presence of a PCR product in lane U indicates unmethylated *LRRC4*. The presence of a PCR product in lane M indicates the methylated *LRRC4*.

In order to demonstrate a functional association between *LRRC4 *promoter methylation and its gene inactivation, a DNA demethylating agent, 5-Aza-2'-deoxycytidine (5-Aza-dC), was used to treat SF126 and SF767 cell lines. By RT-PCR analysis, *LRRC4 *expression was detected after treatment with 5-Aza-dC in both cell lines (Figure 6A). Moreover, *LRRC4 *expression increased with increasing dosage of 5-Aza-dC. *LRRC4 *expression was highest when induced by 15 μM 5-Aza-dC in SF767 cells, but in SF126 cells *LRRC4 *expression was highest when induced by 10 μM 5-Aza-dC.

To confirm that reactivation of *LRRC4 *mRNA expression in glioma cell lines was caused by demethylation of the *LRRC4 *promoter, methylation-specific PCR was used to detect methylation status changes in the *LRRC4 *promoter in the SF767 and SF126 cell lines after 5-Aza-dC treatment. Figure 6B shows that 5-Aza-dC results in the partial demethylation of *LRRC4*.

## Discussion

Inactivation of tumor-suppressor genes plays an important role in malignant brain tumor formation and progression. Genetic mechanisms such as mutation, deletion, and structural chromosome rearrangement are known to inactivate tumor-suppressor genes. Our previous studies demonstrated that the expression of the *LRRC4 *gene was not only highly specific in brain tissue [26], but also a candidate tumor-suppressor gene that may be involved in the pathogenesis of malignant gliomas. However, no genetic alterations of the *LRRC4 *coding region were found in glioma. Based on these data, we speculated that the absence or down-regulation of *LRRC4 *expression in glioma may be caused by the 5' upstream regulatory sequence.

In the present study, we cloned and characterized 2375 bp (-2475 to -101) of the 5' genomic region of the *LRRC4 *gene, which shows baseline promoter activity. Furthermore, transfection experiments using a series of 5'-deleted constructs demonstrated that the region -835 to -293 is sufficient to mediate maximal promoter activity. This region can also drive eGFP expression in the eGFP-reporter plasmid (data not shown). These results suggest that we have found a function of the *LRRC4 *promoter.

The functional *LRRC4 *promoter region is a TATA- and CATT- less, high GC content region and has characteristics of a CpG island. This is consistent with brain-specific gene *LRRC4 *since many tissue-specific genes possess CpG islands in regulatory regions [27].

It is well known that aberrant methylation of CpG islands is one of the major modes of inactivation of tumor suppressor genes in cancer, and a growing list of genes are being identified as abnormal methylation of promoters having CpG islands [28]. Here, the *LRRC4 *promoter was found to be methylated in two glioma cell lines (SF126 and SF767) and all 30 primary gliomas that we have collected, but not in the normal brain tissue samples, suggesting that *LRRC4 *methylation is a tumor-specific event. Furthermore, there was no correlation between clinical stage, sex or age and *LRRC4 *methylation. Methylation of *LRRC4 *was detected in both the early and late stages of glioma, indicating that the inactivation of the *LRRC4 *gene might be essential in the early development of glioma and persist through the course of development.

Treatment of SF126 and SF767 cells with 5-Aza-dC restored *LRRC4 *expression, which suggests that aberrant hypermethylation of the promoter is directly responsible for transcription inactivation of its expression in glioma cell lines. The molecular mechanism through which DNA methylation silences gene expression is not fully understood. DNA methylation may directly interfere with the binding of transcription factors, resulting in the transcriptional repression of the associated gene [29,30]. In addition, methyl-binding domains containing proteins (MBDs) may bind to areas of dense DNA methylation and recruit histone deacetylases and transcriptional repressor complexes, which is refractory to transcription [31]. Bisulfite sequence analysis of the CpG island around the *LRRC4 *promoter reveals dense methylation of CpG sites in glioma cell lines and tissues compared with non-tumor brain specimens (Figure 5). Taken together, these findings suggest that promoter methylation is an important mechanism in the inactivation of *LRRC4 *in glioma.

The present study implies that methylation-mediated inactivation of *LRRC4 *is involved in the initiation and development of glioma. Since *LRRC4 *promoter methylation is found in glioma but not in the normal brain, it may distinguish tumors from normal tissue and serve as a promising biomarker for diagnosis. Our previous study indicated that *LRRC4 *may be an adhesive protein or/and receptor, and it inhibited glioma invasion and metastasis [32]. Since the ability of invasion and metastasis is closely related to prognosis in glioma, *LRRC4 *methylation may also be a biomarker for prognosis. In addition, our findings demonstrate that methylase inhibitor can reverse *LRRC4 *expression in glioma, and it is possible to restore its function as tumor suppressor gene at some degree. This shows that *LRRC4 *may be a potential target for therapy. Of course, all of these hypotheses must be further studied using a large sample analysis.

## Conclusion

In summary, methylation-mediated inactivation of *LRRC4 *is a frequent and glioma-specific event that may be a potential biomarker for diagnosis and prognosis, or a useful target for therapy.

## Competing interests

The authors declare that they have no competing interests.

## Authors' contributions

ZPZ participated in the study design and coordination, data collection, drafting of the manuscript and DNA methylation analysis. DL contributed to patient recruitment, obtaining consent, surgical sample collection and handling, gDNA extraction and DNA methylation analysis. MHW participated in experimental design, helped to draft the manuscript and carried out data interpretation. BX and LW performed the computer analysis of the promoter region and prepared some of the constructs used throughout this work. MZ and PC were involved in cell culture, transfection experiments and analysis and carried out RT-PCR. XLL and SRS participated in drafting and revising the manuscript. GYL carried out the experiment design, manuscript drafting and revision. All authors have read and approved the final version of the manuscript.
